# An Initial Step of GAS-Containing Autophagosome-Like Vacuoles Formation Requires Rab7

**DOI:** 10.1371/journal.ppat.1000670

**Published:** 2009-11-26

**Authors:** Hitomi Yamaguchi, Ichiro Nakagawa, Akitsugu Yamamoto, Atsuo Amano, Takeshi Noda, Tamotsu Yoshimori

**Affiliations:** 1 Structural Biology Center, National Institute of Genetics/SOKENDAI, Mishima-Shizuoka, Japan; 2 Department of Cell Regulation, Research Institute for Microbial Diseases, Osaka University, Suita-Osaka, Japan; 3 Section of Bacterial Pathogenesis, Tokyo Medical and Dental University, Bunkyo-ku, Tokyo, Japan; 4 PRESTO, Japan Science and Technology Agency, Kawaguchi-Saitama, Japan; 5 Department of Cell Biology, Faculty of Bio-Science, Nagahama Institute of Bio-Science and Technology, Nagahama-Shiga, Japan; 6 Department of Oral Frontier Biology, Osaka University Graduate School of Dentistry, Suita-Osaka, Japan; 7 CREST, Japan Science and Technology Agency, Kawaguchi-Saitama, Japan; University of New Mexico, United States of America

## Abstract

Group A streptococcus (GAS; *Streptococcus pyogenes*) is a common pathogen that invades non-phagocytic human cells via endocytosis. Once taken up by cells, it escapes from the endocytic pathway to the cytoplasm, but here it is contained within a membrane-bound structure termed GAS-containing autophagosome-like vacuoles (GcAVs). The autophagosome marker GFP-LC3 associates with GcAVs, and other components of the autophagosomal pathway are involved in GcAV formation. However, the mechanistic relationship between GcAV and canonical autophagy is largely unknown. Here, we morphologically analyzed GcAV formation in detail. Initially, a small, GFP-LC3-positive GcAV sequesters each streptococcal chain, and these then coalesce into a single, large GcAV. Expression of a dominant-negative form of Rab7 or RNAi-mediated knockdown of Rab7 prevented the initial formation of small GcAV structures. Our results demonstrate that mechanisms of GcAV formation includes not only the common machinery of autophagy, but also Rab7 as an additional component, which is dispensable in canonical autophagosome formation.

## Author Summary

Autophagy has become one of the leading edge subjects in science. Autophagy occurs when a cell eats some of its cellular components and digests them. These cellular components may include cytosol and organelles as well as bacteria that has invaded the cell. Thus, autophagy plays an important role in killing pathogens. Here, we introduce an anti-bacterial autophagy called xenophagy. Group A *Streptococcus* (GAS) enters HeLa cells and escapes from the endosome into the cytoplasm for its growth. However, autophagy kicks in and traps GAS, thus preventing its survival path. Detailed morphological observation of this process reveals several specific features which were not found in canonical autophagy. These results provide key information about not only anti-bacterial autophagy, but also canonical autophagy.

## Introduction

*Streptococcus pyogenes*, also known as Group A *Streptococcus* (GAS) is a common pathogen that causes a variety of acute infections including pharyngitis, skin infections, acute rheumatic fever and life-threatening necrotizing fasciitis [1]. The bacterium enters non-phagocytic human cells via endocytosis and subsequently escapes the endolysosomal pathway by inserting bacteria-derived streptolysin O, a cholesterol-dependent pore-forming cytolysin, into the host endosomal membrane [2]. Following bacterial escape into the cytoplasm, GAS is engulfed by a unique structure named GAS-containing autophagosome-like vacuoles (GcAVs) [2]. GcAVs acquire lysosomal enzymes leading to GAS degradation. When GAS release from cells is blocked with tannic acid treatment, the number of organisms recovered from wild-type (WT) cells is reduced by 80%, but there is no reduction in the number of bacteria isolated from autophagy-deficient cells.

Macroautophagy, hereafter referred to simply as autophagy, is a highly conserved cellular process induced by nutrient-starvation that transfers some cytoplasmic components to lysosomes for degradation and recycling of constituent macromolecules [3]. Autophagy involves the formation of a specialized membrane structure, the autophagosome, which is a spherical structure enclosed by two lipid bilayers [4]. The GcAV is considered an “autophagosome-like” structure because of several characteristics shared with autophagosomes. Both structures are labeled with the auophagosomal marker GFP-LC3 [2]. LC3 is the mammalian homologue of yeast Atg8, one of over 30 autophagy-related Atg proteins identified in yeast [5]. Its carboxy terminus is conjugated to the lipid phosphatidylethanolamine by a ubiquitination-like reaction, and this modification leads to LC3 localization on the autophagosomal membrane [6]. Additionally, the formation of both structures depends on Atg5, another protein essential for autophagy [7]. In Atg5 knockout cells, few GcAV are formed [2]. Recently, we showed that the protein complex containing Atg5, Atg12 and Atg16L acted as an E3-like enzyme during LC3 lipidation [8]. We hypothesize that both GcAV formation and autophagy require this protein complex.

Despite these similarities, there are several important differences between autophagosomes and GcAVs. The diameter of autophagosomes is 0.5 to 1.0 µm, but GcAV measure nearly 10 µm across [2]. Furthermore, majority of canonical autophagy is currently thought to non-selectively take up cytosolic components, but GcAV formation is highly specific for GAS sequestration. Thus, although they share some common mechanisms of formation, these two processes are distinct physiologic phenomena.

The autophagic machinery has recently been implicated to play a role in several host-pathogen interactions and host defense which included *Toxoplasma gondii*, *Listeria, Mycobacterium tuberculosis*, and *Shigella* [9],[10],[11],[12],[13]. *Mycobacterium tuberculosis* has the ability to survive within the phagosomal compartment by interfering with phagosome maturation in *Mycobacterium*-containing vacuoles (MCVs), but the autophagic pathway can deliver MCVs to the lysosomal degradative pathway for eventual pathogen elimination [11]. Additionally, ligation of a Toll-like receptor (TLR) at the cell surface leads to its internalization and subsequent recruitment of the autophagic machinery to TLR-containing phagosome [14]. However, the dynamic processes regulating vesicular trafficking and membrane fusion during GcAV formation are largely undefined. In this paper, we morphologically characterize GcAV formation and identify several mechanistic events that occur during GcAV biogenesis. In particular, we show that Rab7 is required for the early phase of GcAV formation. Based on these findings, we develop a membrane dynamic model of GcAV formation and its relation to canonical autophagy.

## Results

### Small GcAVs coalesce to form a large, terminal GcAV

**Figure 1:**
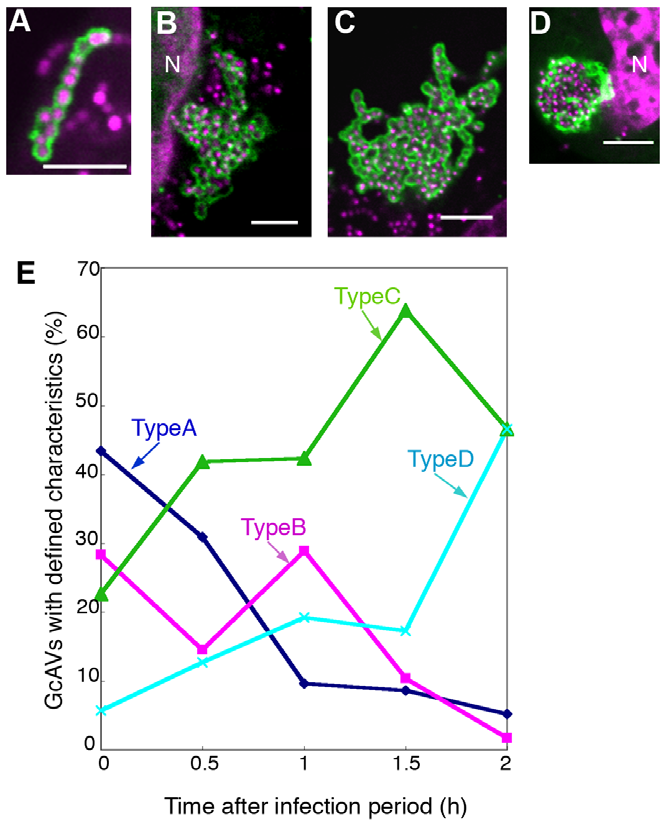
Time-dependent morphological changes in GcAVs. HeLa cells expressing GFP-LC3 were incubated with GAS for 1 h and fixed at 0, 0.5, 1, 1.5, and 2 h after infection, followed by DAPI staining to visualize GAS. The percentage of GcAVs with a given morphological feature was quantified. The defined categories are as follows: (A) type A, a linear chain-like structure (B) type B, an aggregated structure with a clear GFP-LC3 boundary between each cocci; (C) type C, an aggregated structure with a partially discontinuous inner boundary; (D) type D, a semi-spherical structure without a clear inner boundary. Green, GFP-LC3; Magenta, DAPI. N, nuclear. Scale bars, 5 µm. (E) The values are expressed relative to the total number of GcAVs at each time point. Blue, Type A; Pink, typeB; Green, TypeC; Cyan, typeD. The results shown are representative of three independent experiments. Approximately 60 cells were analyzed per experimental group at each time point.

**Figure 2:**
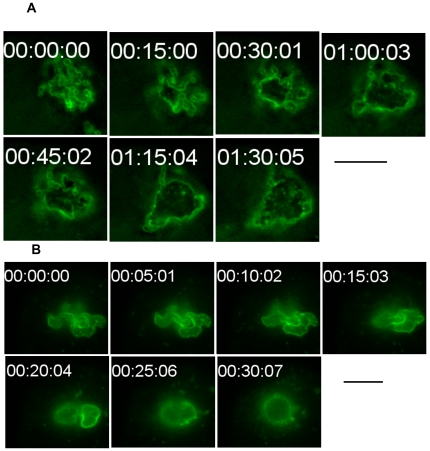
GcAV morphological changes were traced with GFP-LC3. Sequential frames (A, 15-min intervals; B, 5-min intervals) of GcAV formation. HeLa cells expressing GFP-LC3 were incubated with GAS and observed by time-lapse video microscopy. A supplemental video of GcAVs is found online (Video S1, 30-sec intervals). Bars, 10 µm.

**Figure 3:**
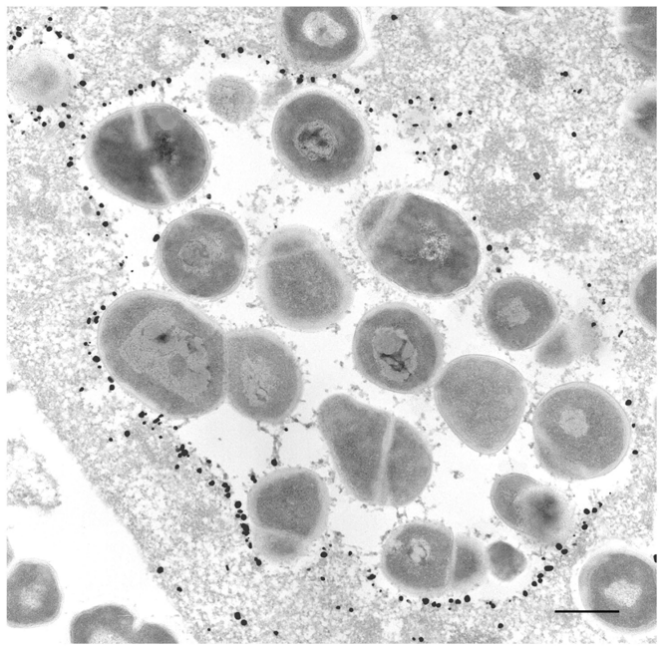
Immunoelectron microscopy for GFP-LC3. After a 2-h infection with GAS, HeLa cells expressing GFP-LC3 were fixed, and the localization of GFP-LC3 was examined by silver-enhanced immunogold electron microscopy using anti-GFP antibodies. GFP-LC3 was predominantly located on membrane compartments containing GAS, but a few dots of GFP-LC3, which were not associated with any membranes, were present on the lumenal side of the compartment. Bar, 0.5 µm.

GAS internalized into HeLa cells eventually come to be contained within membrane delimited intracellular structures called GcAVs [2]. We wished to morphologically characterize GcAV formation in greater detail. HeLa cells expressing GFP-LC3 were infected with GAS, fixed 0.5 h after infection, and examined by fluorescence microscopy. A number of different GcAV morphologies were seen that we subcatergorized into four types as follows (Figure 1A–D): type A, a linear chain-like structure; type B, aggregates of GAS-containing vacuoles with a clear GFP-LC3 boundary between each cocci; type C, a larger aggregate of GAS-containing structures with a partially discontinuous inner boundary; and type D, a semi-spherical structure lacking a clear inner boundary. The frequencies of these different structures changed as the time of infection increased. At 0 h, type A was the most frequently observed, but the number of type A structures decreased with time. Conversely, type D structures increased with time from less than 10% of GAS containing structures to nearly 50%. The observed frequencies of structure types B and C were intermediate, suggesting that these morphologic forms are developmental intermediates (Figure 1E). To confirm this hypothesis, we performed time-lapse imaging of the membrane dynamics in living cells. Several linear chain-like GcAVs (type A) assembled into a collection of grape-like GcAVs (types B), and the boundary between the GAS chains then gradually disappeared (Figure 2A). Indeed, as seen in Video S1, two GAS-containing structures fused to form a larger structure with the appearance of type C structures. We next performed immunoelectron microscopy to analyze the nature of structure type D in greater detail (Figure 3). HeLa cells expressing green fluorescent protein (GFP)-LC3 were infected with GAS and after a 2-h infection, cells were fixed and subjected to immuno electron microscopy (EM) analysis using anti-GFP. GAS was surrounded by single membrane labeled with anti-GFP. Furthermore, these images show that the membrane encompassing the smaller GcAVs is lost upon formation of the larger structures. Taken together, these results indicate that GcAV formation is mediated by the fusion of GAS-containing structures into large, membrane-enclosed compartment.

### Dominant-negative Rab7 inhibits GcAV formation

**Figure 4:**
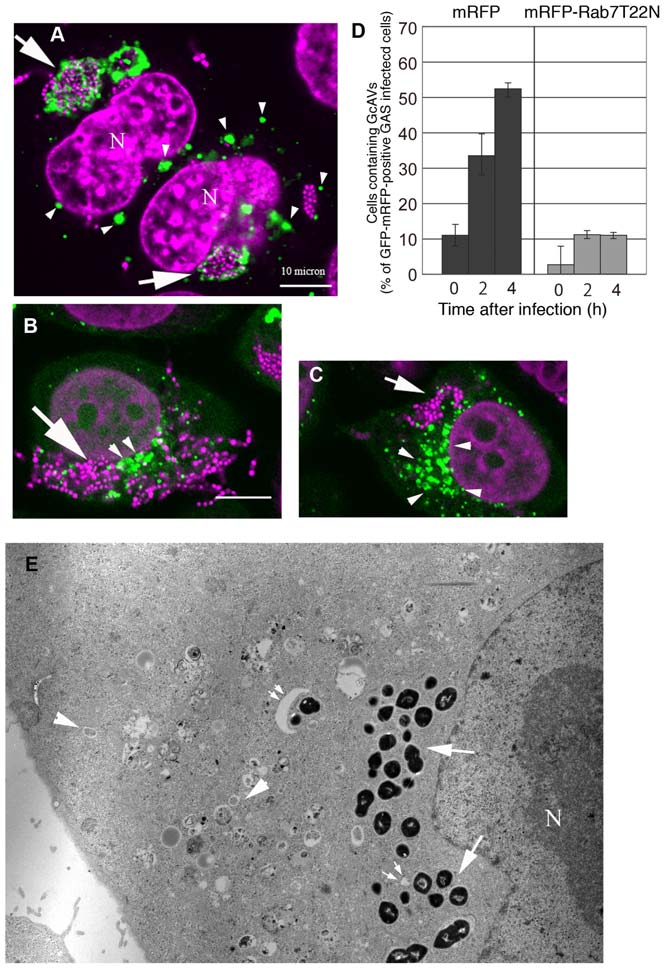
Overexpression of Rab7(T22N) inhibits GcAV formation. HeLa cells expressing GFP-LC3 were transfected with mRFP-Rab7(T22N) (dominant-negative type Rab7) or control mRFP and cultured overnight. Cells were fixed after infection with GAS and stained with DAPI to visualize GAS. (A) Control mRFP-transfected cells. Arrows indicate GcAVs and arrowheads indicate autophagosomes with standard diameters. (B and C) In cells transfected with mRFP-Rab7(T22N), autophagosomes of normal size accumulated (arrowheads), but GcAVs were rarely seen. Green, GFP-LC3; Magenta, DAPI. N, nucleus. (D) The number of cells containing GcAVs was quantified at 0, 2, and 4 h after infection. The cell numbers are expressed as the relative amount compared to the total numbers of GFP- and mRFP-positive GAS-infected cells. Data are means and ranges from three independent assays. Approximately 50 cells were analyzed per experimental group at each time point. (E) An electron micrograph of GAS infected HeLa cells expressing mRFP-Rab7(T22N) 2 h after incubation with GAS. Although immature autophagosomes accumulated in the cytoplasm (arrowheads), GAS were not surrounded by any compartments (arrows). Infrequently, isolation membranes were observed close to the cytoplasmic GAS (double arrows). N; Nuclear.

Our results suggest that homotypic membrane fusion may be involved in GcAV enlargement. We hypothesized that Rab7, a member of the small GTPase Rab family, may be involved in GcAV formation because Ypt7, the yeast orthologue of Rab7, catalyzes the homotypic fusion of vacuoles/lysosomes [15]. To investigate the role of Rab7 in GcAV formation, we used a Rab7 mutant that is constitutively GDP bound and acts as a dominant-negative during the later stages of endocytosis in mammalian cells [16]. HeLa cells expressing GFP-LC3 were transiently transfected with either the dominant-negative Rab7 (T22N) tagged with monomeric red fluorescent protein (mRFP) or mRFP alone as a control, cultured overnight, and then infected with GAS. The cells were fixed and examined by fluorescence microscopy at different time points after infection (Figure 4A–C). Consistent with several previous reports [17],[18],[19], canonical autophagosomes accumulated in the cytoplasm of Rab7(T22N)-expressing cells (Figure 4B and C, arrowheads), reflecting the blockage of fusion between the lysosome and autophagosomes possibly formed by constitutive autophagy. We expected decreased formation of large GcAV and an accumulation of small GAS-containing structures in Rab7(T22N)-expressing cells, but there was quite little accumulation of GFP-LC3 signal around GAS, indicating that LC3 recruitment to GcAV or GcAV formation is defected (Figure 4D). GcAv formation was not substantially affected in cells overexpressing WT Rab7 or a GTPase deficient mutant (Q67L) (data not shown). To confirm these observations, we extended our analysis with electron microscopy. As before, there was significant accumulation of canonical autophagosomes within the cytoplasm of Rab7(T22N)-expressing cells after infection with GAS (Figure 4E, arrowheads). However, no GAS were enclosed in GcAV-like membrane delimited structures (Figure 4E, arrows) characterized by canonical autophagosome or isolation membrane like double membrane (Figure 4E, arrowheads and small arrows), indicating that GcAVs were not formed. Thus, we conclude that Rab7 inactivation disrupts an early step in GcAV formation.

**Figure 5:**
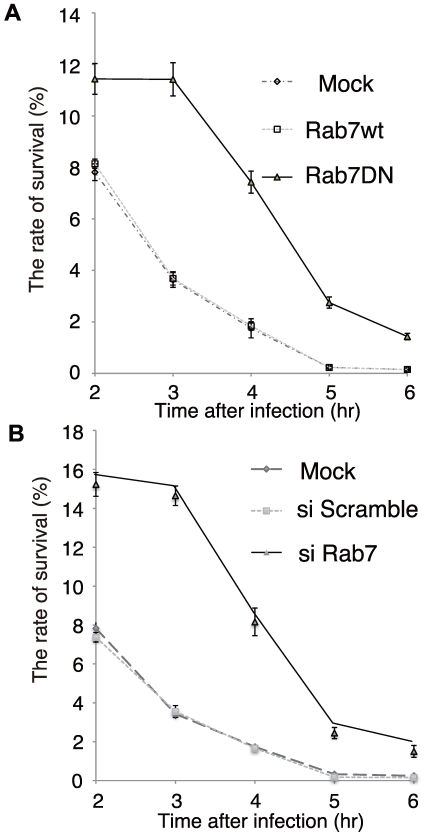
Effects of Rab7 on the viability of intracellular GAS in HeLa cells. (A) HeLa cells were infected with adenovirus encoding a dominant-negative form of Rab7 (Rab7DN-Ad) or WT Rab7. Two days after infection, HeLa cells and adenovirus-infected cells were infected with GAS for 1 h. The numbers of intracellular live GAS were determined with a colony forming assay and presented as ratio of “intracellular live GAS at the indicated time” to “intracellular and adhered GAS at 1 h”. To suspend the escape of intracellular GAS, cells were treated with 0.5% tannic acid (TA) twice. (B) Rab7-RNAi or negative control (scramble) RNAi were transfected into HeLa cells. Two days after transfection, HeLa cells and RNAi transfected HeLa cells were infected with GAS for 1 h, and the intracellular number of viable GAS were determined by colony counting and presented as ratio of “intracellular live GAS at the indicated time” to “intracellular and adhered GAS at 1 h”. To suspend the escape of intracellular GAS, cells were treated twice with 0.5% TA.

To further define the role of Rab7 in GcAV formation, we examined the effects of Rab7 inactivation on the killing of GAS within GcAV. HeLa cells were transduced with adenovirus encoding Rab7(T22N), and these cells were then infected with GAS and treated with tannic acid to prevent GAS escape to the cytoplasm [2]. The number of surviving bacteria was counted using a colony formation assay. Expression of Rab7(T22N) decreased the efficiency of endocytic internalization of GAS by 35.9±2.1% (average ± SD of four independent experiments) compared to control cells, possibly reflecting negative feedback within the endocytic pathway. Despite the decreased uptake, however, greater numbers of GAS were recovered from Rab7(T22N)-expressing cells at all time points (Figure 5A). To confirm this finding, we used RNAi to knockdown Rab7 expression in HeLa cells, and virtually identical results were obtained (Figure 5B). Taken together, these results clearly establish a direct requirement for Rab7 in GcAV formation and GAS killing.

### Identification of the GcAV precursor structure

**Figure 6:**
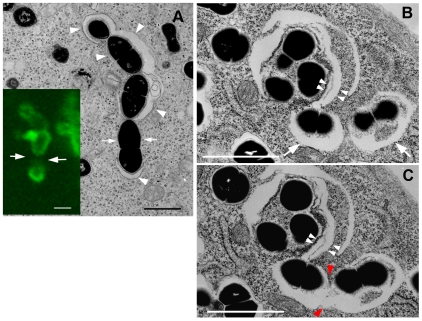
Precursor GcAV structure. (A) HeLa cells expressing GFP-LC3 grown on a gridded glass bottom dish were incubated with GAS for 1 h and followed by incubation in culture medium containing antibiotics for 0.5 h. An EM image shows several isolation membranes (arrowheads) surrounding cytoplasmic GAS. A correlative confocal image showed a discontinuous GFP-LC3 pattern (lower left panel, green). Arrows indicate gaps between individual isolation membranes. (B and C) Pictures represent serial sections of the same cell 0.5 h after infection. Two individual isolation membranes enwrapping GAS (B, arrows) fused with each other at the point indicated by red arrowheads (C). Double arrowheads indicate overlapping isolation membranes around GAS. Bars, 1 µm (A–C).

To better understand the mechanism(s) regulating GcAV formation, we next wished to identify the GcAV precursor structure. We used correlative fluorescence and electron microscopy to examine HeLa cells expressing GFP-LC3. After GAS infection and fixation, a GFP-LC3 positive structure, morphologically distinct from types A, B, C, or D described above, was identified incompletely surrounding several bacteria by fluorescence microscopy. By electron microscopy, this corresponded to a sac-like structure aligned along the GAS bacteria at the sites of GFP-LC3 signal (Figure 6A). To better understand the spatial relationship of this sac-like structure, we performed serial sectioning and electron microscopy. In Figure 6B and C, two membrane sacs appear associated with the same bacterium (white arrowheads). Moreover, successive electron microscopic images revealed that a double-membrane bound structure encompassing a GAS bacterium in one area (Figure 6B, white arrows) was connected to another such structure in the next section (Figure 6C, red arrowheads).

**Figure 7:**
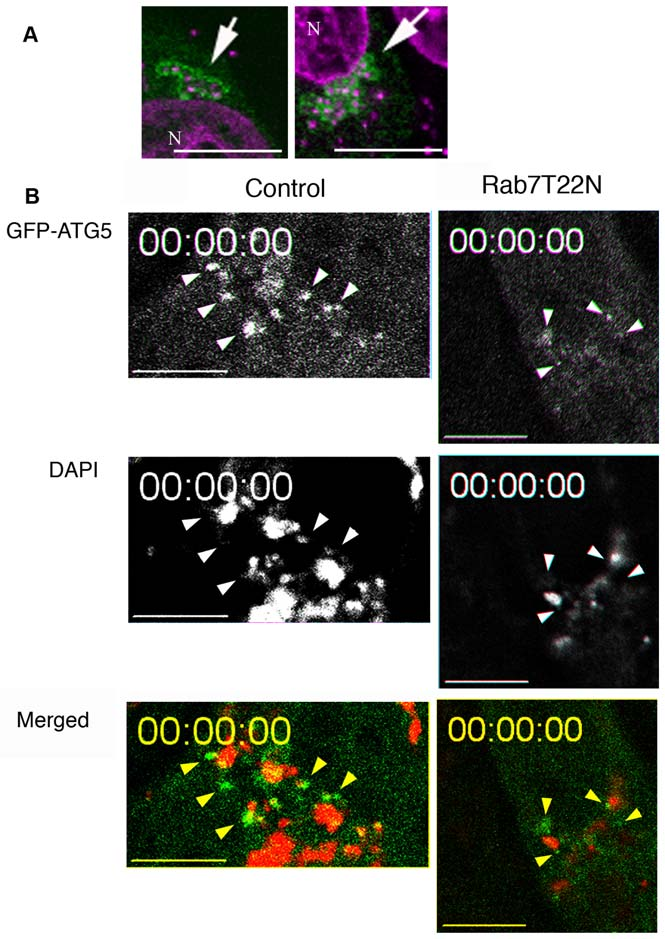
Localization of GFP-Atg5. (A) After 0.5-h infection with GAS, HeLa cells expressing GFP-Atg5 were fixed and stained with DAPI. GFP-Atg5 signals were observed around GAS (arrows). Green, GFP-Atg5; Magenta, DAPI. N, nuclear. (B) HeLa cells expressing GFP-Atg5 were transfected with mRFP-Rab7(T22N) and cultured overnight or cells without transfection (Control) were infected with DAPI-stained GAS. One hour after GAS infection, cells were observed by live-video microscopy. GFP-Atg5 punctate staining close to GAS was observed (arrowheads). Green, GFP-Atg5; Red, DAPI. Also see Videos S2 and S3. Bars, 10 µm.

Atg5 is transiently associated with isolation membranes, the intermediate form of autophagosome [7], and we next determined the localization of Atg5 during GcAV formation. When HeLa cells expressing GFP-Atg5 were infected with GAS, GFP-Atg5 was seen around GAS bacteria (Figure 7A). Video microscopy of living cells demonstrated the transient appearance of multiple punctate spots of GFP-Atg5 near individual bacteria (Figure 7B control and Video S2). These spots were highly mobile, suggesting that they correspond to individual membrane-bound structures, but not specialized domain in continuous membrane. These results support the model that multiple, smaller membrane-bound structures surround a GAS chain and coalesce to form GcAVs.

**Figure 8:**
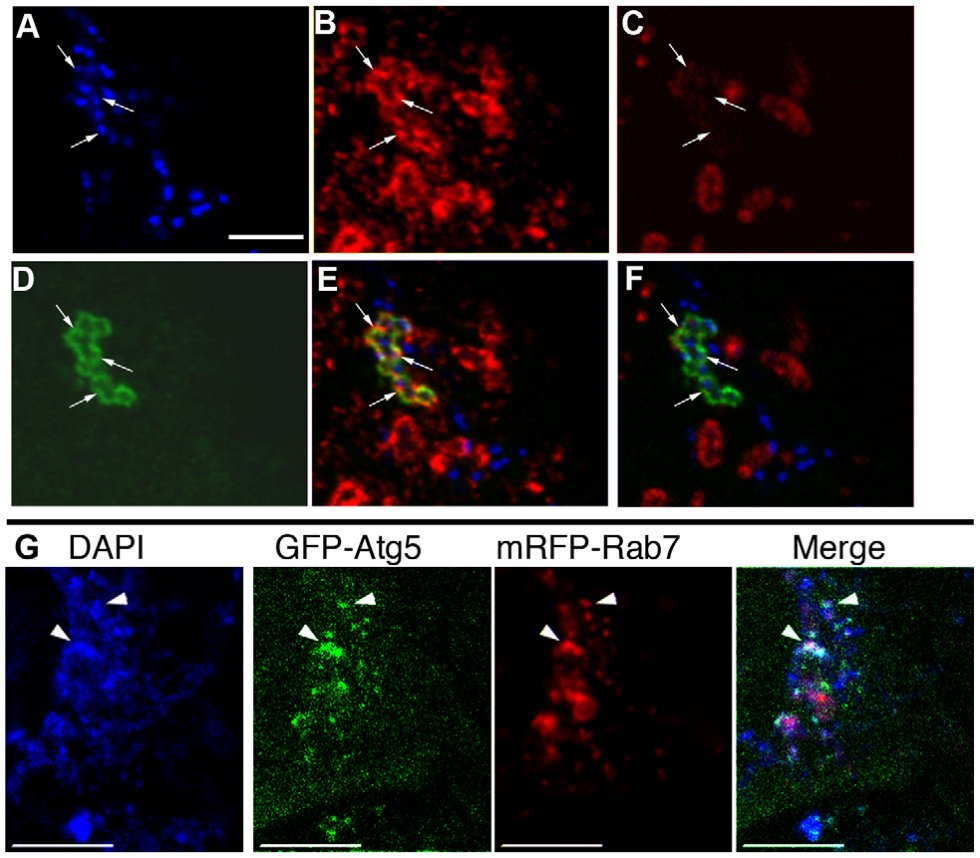
Rab7 localizes to isolation membranes and GcAVs in the early phase. NIH3T3 cells transfected with GFP-LC3 were fixed 0.5 h after GAS infection. Images (A–F) show fluorescence micrographs of the same field. (A) DAPI staining, (B), Rab7 staining (C) Lamp-1 staining, (D) GFP-LC3 labeling, (E) a merged image of (A) (B) (C), (F) a merged image of (A) (B) (D). Arrows indicate Rab7-positive but Lamp-1 dim GcAVs (G) mRFP-Rab7 was recruited to GFP-Atg5 positive compartments adjacent to GAS. HeLa cells expressing GFP-Atg5 and mRFP-Rab7 were observed by fluorescence microscopy 1 h after GAS infection. mRFP-Rab7 was often observed to colocalize with GFP-Atg5 at DAPI-stained GAS (arrowheads). Bars, 10 µm.

In cells expressing Rab7(T22N), GFP-Atg5 positive structures were also observed adjacent to GAS (Figure 7B Rab7(T22N), Video S3), and, when the number of contacts between GAS and GFP-Atg5 was quantified, 26 (RFP) and 27 cells (T22N) contained such sites of contacts (Table 1). We next examined the localization of endogenous Rab7. NIH3T3 cells expressing GFP-LC3 were infected with GAS, and the cells were fixed and processed for immunofluorescent confocal microscopy using anti-Rab7 antibodies. In these cells (Figure 8A–F), Rab7 localized to GcAVs labeled by GFP-LC3. Rab7 localizes to late endosomes and lysosomes [16], and, because GcAVs terminally fuse with lysosomes [2], the colocalization of Rab7 with GFP-LC3 positive GcAVs is not entirely unexpected. However, as shown in Figure 8A–F, there is a sub-population of Rab7 that localized to GcAVs that were not well stained by the lysosomal membrane marker, Lamp-1. Observation of live cells using fluorescently labeled Rab7 (mRFP-Rab7) and GFP-Atg5 demonstrated colocalization of mRFP-Rab7 and GFP-Atg5 at DAPI-stained GAS (Figure 8G). Thus, a population of Rab7 is recruited to GFP-Atg5 positive membranes during the early phase of GcAV formation. These results indicate that Rab7 plays an important role in the early phase of GcAV formation.

**Table 1:** A summary of fluorescence observation of the cells expressing mRFP or mRFP-Rab7T22N.

	Control mRFP expressing cell	mRFP-Rab7T22N expressing cell
Cells bearing GFP-Atg5 punctate spots (%)	55.3	64.2
Number of GFP-Atg5 spots Per cell	2.4	2.1
Spots emerging adjacent to GAS (%)	58.3	48.6
Spots emerging in GAS free region (%)	41.7	51.4

## Discussion

In this study, we mechanistically characterized GcAV formation and demonstrated that this process is biphasic. Each bacterium is enclosed by a membrane and these structures fuse into a membrane-bound structure to form a large spherical GcAV. Rab7 is involved in the early phase of GcAV formation, though it may play a role in later stages as well. The final phase of GcAV formation is the appearance of a semi-spherical large GcAV enclosed by a single membrane. However, because each bacterium is enclosed in a membrane, the generation of a border composed of a single membrane requires the loss of the internal membranes surrounding each GAS. An identical phenomenon is observed during canonical autophagy when the double-membrane lined autophagosome is converted into the single membrane enclosed autolysosome. Additionally, as in canonical autophagy, the inner membrane of the GcAV becomes degraded following the acquisition of lysosomal contents including lipases. Indeed, we previously showed that GcAVs fused with lysosomes during the late stages of their formation [2]. Thus, GcAV formation is a highly complex process involving dynamic membrane rearrangements. It is likely that the physiochemical properties including associated proteins govern the size and structure of the terminal GcAV, but the precise factors governing this structure are unknown.

We identified the precursor structure of the GcAV, and it is a membrane-bound organelle very similar to the isolation membrane of canonical autophagy. Both structures are labeled with LC3 and Atg5, and, although the exact composition of the isolation membrane remains unclear, it is possible that these two structures are identical or, at the very least, share a number of common lipid and protein components.

One key difference between GcAV formation and canonical autophagy, however, is the specificity of the process, and it is likely that GAS recognition factors are incorporated into the nascent GcAV. Additionally, multiple membrane bound structures are recruited to a GAS chain, and they coalesce to form a larger structure encompassing all of the bacteria. The recruitment and fusion of these vesicles into a larger GcAV may proceed without specific adaptor molecules, or, conversely, another organelle (e.g. lysosomes) may mediate the fusion of two or vesicles simultaneously. In either case, the membrane fusion event is similar to that leading to autophagosome formation. In autophagy, isolation membranes undergo two distinct fusion events: the closing of the isolation membrane during autophagosome formation, and the fusion between autophagosome and lysosome. Therefore, both of our alternative hypotheses are possible and consistent with events known to occur during autophagy. Indeed, we occasionally observed two morphologically distinct membrane structures connected in serial EM sections, consistent with ongoing fusion events.

Interestingly, inactivation of Rab7 impaired GcAV formation and as a result, intracellular survival of GAS increased. The impact of Rab7 inactivation to the GAS killing is milder than that of the Atg5 knock out, but it may be due to impairment of other cellular process because Rab7 is also involved in broad range of physiological responses through the regulation of endocytosis. Or in the presence of intact canonical autophagosome dependent protein sequestration, some unknown bacterial killing system other than autophagy might be induced to compensate for GcAV function. Members of the Rab family including Rab7 are involved in membrane fusion events throughout the secretory and endocytic pathways. During GcAV formation, however, the pre-fusion intermediate structures did not accumulate in cells expressing the Rab7 dominant-negative mutant. Thus, Rab7 may be necessary for the generation of the precursor membrane structure. However, we observed comparable amounts of GFP-Atg5 associated with GAS in cells expressing the Rab7 dominant-negative mutant, and this event presumably occurs later in GcAV formation. In canonical autophagy, Rab7 is not required for autophagosome formation, but it is essential for the fusion of lysosomes and autophagosomes [17],[18]. Consequently, if GcAV formation largely recapitulates autophagy, the role of Rab7 in the early stages of GcAV formation may be more complicated than simply mediating precursor formation. Alternatively, Rab7 may be involved in the stabilization of precursor vesicles and/or its delivery to GcAV immediately prior to their association with GAS. The presence of Rab7 on GAS-containing structures during the early phases of GcAV formation is consistent with this possibility. We should also stress the possibility that Rab7 is also involved in the later steps of these processes, such as fusion of isolation membranes, homotypic fusion of small GcAV, fusion between GcAV and lysosome, although our experimental system was unable to directly demonstrate the defects in these subsequent events. Further analyses of the composition and formation of GcAV and all precursor structures will help refine these models.

In this report, we have further characterized the morphologic features and membrane fusion events associated with GcAV formation. We demonstrated that GcAV formation, while similar to canonical autophagy, has several unique features. The GcAV pathway likely represents a Rab7-dependent evolutionary variation on canonical authophagy to sequester and degrade microorganisms that have evolved strategies to escape from lysosomal degradation. Further studies will identify the mechanism of not only GcAV formation but also of canonical autophagy.

## Materials and Methods

### Bacterial strains

GAS (*S. pyogenes*) strain JRS4 (M6+ F1+) was grown in Todd-Hewitt broth (BBL, Cockeysville, MD, USA) supplemented with 0.2% yeast extract (THY) as described previously [20].

### Cell culture and transfection

HeLa cells, HeLa cells stably expressing GFP-LC3 [5] or GFP-Atg5 [7], NIH3T3 cells and 293A (Invitrogen) were maintained in DMEM [Dulbecco's modified Eagle's medium (DMEM, Sigma-Aldrich, MO, USA) containing 9% fetal bovine serum (FBS, Invitrogen, CA, USA) and 2 mM L-glutamine (Invitrogen)] in a 5% CO_2_ incubator at 37°C. Transfection was done with LipofectAmine2000 reagent (Invitrogen) according to the manufacturer's instructions. To obtain stable transformants, cells were selected in the presence of 0.5 mg/ml G418. For knock-down of Rab7, RNAi targeting human Rab7 (CGGTTCCAGTCTCTCGGTGTT; corresponding to human Rab7 cDNA of 205–225 bp) and siPerfect Negative control RNAi (Sigma Genosys) were transfected using jetPEI (Polyplus-transfeciton, Inc). For transient expression of the Rab7(T22N) mutant by adenovirus-mediated transfer, ViraPower adenoviral Gateway expression kit (Invitrogen) was used according to the manufacturer's instructions.

### Infection protocol

For confocal microscopy analysis, cells (8×10^4^ cells) were seeded in 500 µl of medium in 24-well tissue culture plates containing 12-mm-diameter glass coverslips. After cells were grown overnight, the spent medium was removed and replaced with fresh medium. Bacteria were harvested and washed twice with DMEM, then were added to cell cultures at a multiplicity of infection (MOI) of 100 (cells:bacteria = 1∶100), without antibiotics. After incubation with GAS for 1 h, cells were washed twice with DMEM to remove nonadherent bacteria, and cells were further incubated for the indicated times in DMEM/FBS containing antibiotics [100 µg/ml of gentamicin (Sigma) and 100 µg/ml of streptomycin (Invitrogen)] to kill extracellular bacteria. For live cell imaging, GAS bacteria were stained with 0.4×10^−2^ ng/ml DAPI (4′,6-Diamidino-2-phenylindole dihydrochloride, Sigma) for 15 min before infection.

### Bacterial viability assay

HeLa cells (2×10^4^ cells/well) were cultured in 24-well culture plates. Transfection of Rab7-RNAi or negative control of RNAi (20 pmol) or adenovirus-mediated transfer (moi = 100) were performed 48 h before infection experiments. Cells were infected as described above. To prevent the intracellular release of GAS, 0.5% tannic acid was added to the medium twice, both at 15 min before antibiotic treatment and at 2 h after infection. After an appropriate incubation time, infected cells were lysed in sterile distilled water and serial dilutions were plated on THY agar plates. The number of viable intracellular bacteria was determined and presented as the ratio of “intracellular live GAS at the indicated time” to “intracellular and adhered GAS at 0 h”, with ± s.e for six independent experiments.

### Expression plasmids, antibodies and fluorescent chemicals

LC3 and Rab7(T22N) were fused to the C-terminus of mRFP [21] and transiently expressed under the control of the CMV IE promoter. The point mutation (T22N) was introduced by Quick change system (Invitrogen). For construction of Rab7(T22N) expressing adenovirus vector, the WT Rab7 or Rab7(T22N) fragment were cloned into pENTR-D-TOPO, and converted to adenovirus vector according to the manufacturer's instructions (Invitrogen). Rabbit serum targeting Rab7 was a kind gift from Dr. E. Kominami (Juntendo University, Tokyo, Japan). Antibodies were used at the following concentrations: rabbit anti-Rab7, 100x dilution of a stock; mouse monoclonal anti-human Lamp1 (clone H4A3; Santa Cruz Biotechnology, CA, USA), 10 µg/ml; rabbit anti-GFP [7], 1000x dilution of a stock. The secondary antibodies were Alexa568-conjugated anti-rabbit IgG (Molecular Probes, OR, USA) and Alexa647-conjugated anti-mouse IgG (Molecular Probes). All secondary antibodies were diluted 1∶400. To label bacterial and cellular DNA, cells were stained with 1 µg/ml DAPI dissolved in PBS.

### Immunofluorescence confocal microscopy

For Immunofluorescence microscopy, NIH3T3 cells expressing GFP-LC3 were infected with GAS for 0.5 h and pre-permeabilized for 5 min in PBS containing 50 µg/ml digitonin. The cells were then washed once in PBS and fixed for 15 min in 4% paraformaldehyde. The cells were then blocked in PBS containing 0.1% gelatin (gelatin-PBS) for 0.5 h, followed by incubation with primary antibodies diluted in gelatin-PBS for 2 h. After the cells were washed six times in PBS (5 min for each), they were incubated with the secondary antibodies diluted in gelatin-PBS for 1.5 h and washed six times in PBS (5 min for each). All steps were done at room temperature. The cells were then mounted with Slow Fade (Molecular Probes) and observed under a fluorescence laser-scanning microscope (FV1000, Olympus, Japan).

### Quantification of cells bearing GcAVs and morphological study of GcAVs

HeLa cells expressing GFP-LC3 were transfected with mRFP or mRFP-Rab7(T22N), cultured overnight and infected with GAS. At 0, 2, and 4 h after the infection period, cells were fixed and stained with DAPI. The number of cells containing GcAVs was quantified at each time point. Transfected cells were identified by mRFP fluorescence. The rate of cellular GcAV formation was expressed as a percentage of the total LC3-, mRFP-positive and GAS-infected cells. Approximately 50 cells were analyzed in each assay. For morphological analysis, HeLa cells expressing GFP-LC3 were incubated with GAS and observed at the indicated time points. The ratio of each morphologically characterized GcAV was calculated as a percentage of total observed GcAVs. Approximately 60 cells were analyzed at each time point.

### Fluorescence microscopy and quantification of cells with punctate GFP-Atg5

HeLa cells expressing GFP-Atg5 were grown, transfected and incubated infected with GAS on glass-bottom culture dishes (Synapse Fine-View dish, SF-T-D12, Ivic, Japan). At the indicated time after infection, cells were analyzed by video microscopy at 37°C on a IX81 (Olympus) equipped with a cooled CCD camera (Roper Scientific, Japan). Time-lapse recording was operated with the SlideBook Imaging System (Intelligent Imaging Innovations. Inc., CO, USA). To observe live cells, we used a CO_2_ incubator (MI-IBC, Olympus) that allows accurate control of the microenvironment. The recorded images were processed using Image J software. The presented images are representative of approximately 45 cells from at least 8 independent experiments. The percentage of cells with GFP-Atg5 punctate dots was quantified as follows: During 1- to 2-h period after infection with GAS, the cells expressing GFP-Atg5 and mRFP/mRFP-Rab7(T22N) were routinely recorded with a 1-min interval for 20 min. We scored cells in which GFP-Atg5 staining colocalized with or contacted DAPI-stained GAS in at least 2 successive frames. For individual dots, we sorted them according to whether or not the dot would colocalize with/contact GAS, when the dot first appeared in the frame.

### Correlative FM-EM and conventional EM

For Correlative FM-EM, cells expressing GFP-LC3 were cultured on uncoated glass bottom culture dishes with grid patterns (Mat Tek, Ashland, MA, USA) in 1 ml DMEM. At 0.5 or 1 h after infection, an equal volume of the first fixative [1.25% glutaraldehyde (Distilled EM grade, EM sciences, PA, USA) and 1% formaldehyde (EM grade, Nacarai, Japan) dissolved in 0.1 M cacodylate buffer (pH, 7.4)] was added, followed by 5-min incubation. After the fixative was aspirated, the cells were subsequently incubated with the second fixative (1.25% glutaraldehyde and 1% formaldehyde dissolved in the same buffer) for 1 h. The cells were further incubated with the third fixative (2.5% glutaraldehyde and 2% formaldehyde dissolved in the same buffer) for 4 h. All fixations were performed at room temperature. The fixed specimens were washed three times with 0.1 M cacodylate buffer (pH, 7.4) containing 7% sucrose at 4°C (5 min for each). Then, the cells bearing GcAVs were identified and photographed by phase-contrast and fluorescence microscopy. Next, the samples were fixed with 1% osmium dissolved in cacodylate buffer and block-stained in 0.5% uranyl acetate. After dehydration in a graded ethanol series, they were embedded in Epon 812 resin (TAAB, UK). Ultra thin sections were double-stained with uranyl acetate and Reynolds' lead citrate [22], and the cells of interest were analyzed using a JEOL JEM-1011 transmission electron microscope. For conventional EM, the cells were cultured on collagen-coated plastic coverslips (Sumitomo Bakelite, Japan) in 500 µl DMEM in 24-well tissue culture plates. Subsequent procedures were done as described above.

### Immunoelectron microscopy

After a 2-h infection, HeLa cells expressing GFP-LC3 via adenoviral transduction [2] were fixed with 4% paraformaldehyde and 0.1% glutaraldehyde in 0.1 M Na-phosphate buffer, pH 7.4 for 1 h at room temperature. The pre-embedding silver enhancement immunogold method was performed as previously described [23].

## Supporting Information

Video S1 Morphological progression of GcAVs. HeLa cells expressing GFP-LC3 were cultured on a glass bottom culture dish and were infected with GAS At 2 h after the infection period, the cells were observed by time-lapse video microscopy. Two semi-spherical GcAVs were observed fusing to each other.
(0.26 MB MOV)

Video S2 GFP-Atg5 spots around GAS. HeLa cells expressing GFP-Atg5 were infected with GAS and were observed by time-lapse video microscopy. Green, GFP-Atg5; Magenta, DAPI.
(0.77 MB MOV)

Video S3 GFP-Atg5 spots were observed around GAS in a cell expressing mRFP-Rab7T22N. HeLa cells expressing GFP-Atg5 were transfected with mRFP-Rab7T22N. The cells were observed by time-lapse video microscopy after the infection period. Green; GFP-Atg5, Magenta; DAPI.
(0.96 MB MOV)
